# Hyperoxemia as a risk factor for ventilator-associated pneumonia

**DOI:** 10.1186/s13054-016-1368-4

**Published:** 2016-06-22

**Authors:** Sophie Six, Karim Jaffal, Geoffrey Ledoux, Emmanuelle Jaillette, Frédéric Wallet, Saad Nseir

**Affiliations:** CHU Lille, Centre de Réanimation, F-59000 Lille, France; CHU Lille, Centre de Biologie et de Pathologie, F-59000 Lille, France; Univ Lille, Faculté de Médecine, F-59000 Lille, France

**Keywords:** Arterial oxygen tension, Ventilator-associated pneumonia, Critical care, Prevention, Outcome, Hyperoxemia, Hyperoxia

## Abstract

**Background:**

Consequences of hyperoxemia, such as acute lung injury, atelectasis, and reduced bacterial clearance, might promote ventilator-associated pneumonia (VAP). The aim of our study was to determine the relationship between hyperoxemia and VAP.

**Methods:**

This retrospective observational study was performed in a 30-bed mixed ICU. All patients receiving invasive mechanical ventilation for more than 48 hours were eligible. VAP was defined using clinical, radiologic, and quantitative microbiological criteria. Hyperoxemia was defined as PaO_2_ > 120 mmHg. All data, except those related to hyperoxemia, were prospectively collected. Risk factors for VAP were determined using univariate and multivariate analysis.

**Results:**

VAP was diagnosed in 141 of the 503 enrolled patients (28 %). The incidence rate of VAP was 14.7 per 1000 ventilator days. Hyperoxemia at intensive care unit admission (67 % vs 53 %, OR = 1.8, 95 % CI (1.2, 29), *p* <0.05) and number of days spent with hyperoxemia were significantly more frequent in patients with VAP, compared with those with no VAP. Multivariate analysis identified number of days spent with hyperoxemia (OR = 1.1, 95 % CI (1.04, 1.2) per day, *p* = 0.004), simplified acute physiology score (SAPS) II (OR = 1.01, 95 % CI (1.002, 1.024) per point, *p* < 0 .05), red blood cell transfusion (OR = 1.8, 95 % CI (1.2, 2.7), *p* = 0.01), and proton pomp inhibitor use (OR = 1.9, 95 % CI (1.03, 1.2), *p* < 0.05) as independent risk factors for VAP. Other multiple regression models also identified hyperoxemia at ICU admission (OR = 1.89, 95 % CI (1.23, 2.89), *p* = 0.004), and percentage of days with hyperoxemia (OR = 2.2, 95 % CI (1.08, 4.48), *p* = 0.029) as independent risk factors for VAP.

**Conclusion:**

Hyperoxemia is independently associated with VAP. Further studies are required to confirm our results.

## Background

Hyperoxemia is common in mechanically ventilated critically ill patients. Its incidence ranges from 16–50 % in this population [1–5]. Liberal oxygen therapy is supposed to prevent hypoxia and improve the oxygen supply to the different affected organs. However, evidence from several recent studies suggests that hyperoxemia is probably not safe. Hyperoxemia is responsible for vasoconstriction and decreased cardiac output, resulting in reduced blood flow and oxygen transport [6, 7]. A negative impact of hyperoxemia on mortality is also reported in different patient populations, such as patients undergoing resuscitation from cardiac arrest [8], patients with acute ischemic stroke [5], and patients with ST-segment elevation [9]. In mechanically ventilated patients, the relationship between mortality and hyperoxemia is still controversial [10–12]. The definition of hyperoxemia, measurement methods, and patient population are very heterogeneous in the available studies, making it difficult to interpret the results.

**Fig. 1:**
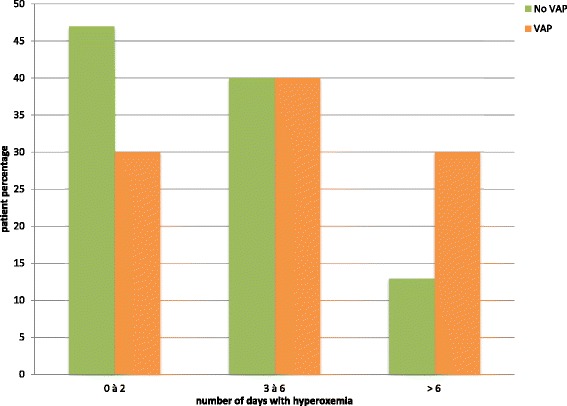
Distribution of ventilator-associated pneumonia (*VAP*) episodes based on number of days with hyperoxemia

Exposure to hyperoxia is responsible for hyperoxic acute lung injury (HALI). Pulmonary edema, hyaline membrane formation, pulmonary arteriole thickening, and alteration in the ventilation/perfusion fraction are the main mechanisms described in HALI [13, 14]. In addition, denitrogenation phenomena, and inhibition of surfactant production are observed at high levels of inspired oxygen fraction (FiO_2_), promoting expiratory collapse and atelectasis [15, 16]. Hyperoxia also impairs mucociliary clearance and the antimicrobial action capacity of macrophages and immune cells [17]. Acute lung injury, atelectasis, and reduced bacterial clearance are well-known risk factors for VAP [18–20].

VAP is the most common ICU-acquired infection, and is associated with high mortality, duration of mechanical ventilation, and cost [21]. Therefore, prevention of VAP is a key issue in critically ill patients. Understanding the pathophysiology of VAP, and identification of risk factors are important in order to improve preventive strategies [22].

Previous animal studies strongly suggest a relationship between hyperoxemia and VAP. However, in spite of the obvious potential link between hyperoxemia and VAP, to our knowledge no study to date has evaluated the relationship between these two conditions. Therefore, we conducted this retrospective observational study to determine whether hyperoxemia is a risk factor for VAP.

## Methods

### Study characteristics

This retrospective observational study was performed in a 30-bed mixed ICU, located in the University Hospital of Lille, France. All data, except blood gases results, were collected prospectively during an 18-month period. Because of the observational design, and in accordance with French law, written informed consent was not required. The local institutional review board (Comité de Protection des Personnes Nord-Ouest IV) approved the study. All patients requiring invasive mechanical ventilation for more than 48 h, during an 18-month period, were eligible. The only exclusion criterion was duration of mechanical ventilation for ≤48 h.

### Definitions

VAP was defined as the presence of new or progressive pulmonary infiltrate, and at least two of the following criteria: (1) fever (body temperature ≥38 °C) or hypothermia (<36 °C); (2) leukocytosis (>12 × 10^9^/L leukocytes) or leukopenia (<3.5 × 10^9^/L); and (3) purulent respiratory secretions. Microbiological confirmation was required in all patients (positive bronchoalveolar lavage ≥10^4^ colony-forming units (cfu)/mL, or positive tracheal aspirate ≥10^5^ cfu/mL) [23]. Only first episodes of VAP diagnosed >48 h after starting mechanical ventilation were taken into account. Early-onset episodes were defined as VAP diagnosed <5 days after starting mechanical ventilation, and late-onset episodes as VAP diagnosed ≥5 days after mechanical ventilation. Hyperoxemia was defined as arterial oxygen tension (PaO_2_) >120 mmHg. Arterial blood gases were analyzed on a daily basis, and each day with at least one PaO_2_ value >120 mmHg (>16 kPa) was considered as a day with hyperoxemia.

The following microorganisms were defined as multidrug-resistant bacteria: ceftazidime or imipenem-resistant *Pseudomonas aeruginosa*, *Acinetobacter baumannii*, *Stenotrophomonas maltophilia*, β-lactamase-producing Gram-negative bacilli and methicillin-resistant *Staphylococcus aureus*. Prior antibiotic use was defined as antimicrobial treatment during the 3 months preceding ICU admission. Immunosuppression was defined by the presence of neutropenia (neutrophils <500/μL), active solid or hematological malignancy, long-term corticosteroid therapy (≥1 mg/kg/d for >1 month), or HIV infection (CD4 < 50/μL during the previous 6 months).

### Study patients

A VAP prevention strategy was routinely used during the study period. The ventilator circuit was not changed routinely. In all patients, a heat-moisture exchanger was positioned between the Y piece and the patient. These exchangers were changed every 48 h or more frequently if visibly soiled. Sedation and weaning were based on a written protocol. A minimal positive end-expiratory pressure of 5 cm H_2_O was used in all patients. Selective digestive decontamination and subglottic secretion drainage were not used. The oral cavity was cleaned with chlorehexidine three times daily. Cuff pressure was measured and adjusted (25 cm H_2_O) by nurses thrice a day. Tracheal suctioning was routinely performed by nurses, using an open tracheal suction system. Patients remained in the semi-recumbent position, and received enteral nutrition based on a written protocol.

Antibiotic treatment for patients with suspected VAP was based on American Thoracic Society/Infectious Diseases Society of America (ATS/IDSA) guidelines [23]. Antibiotic treatment for other infections was based on written local guidelines adapted from international and national guidelines. Stress ulcer prophylaxis was not routinely used.

### Data collection

All data were prospectively recorded, except blood gases results. The following characteristics were recorded at ICU admission: age, male gender, severity of illness based on simplified acute physiology score (SAPS) II, logistic organ dysfunction (LOD) score, and McCabe score; comorbidities (diabetes, chronic obstructive pulmonary disease (COPD), chronic heart failure, cirrhosis, chronic renal failure requiring dialysis, or immunosuppression), location before ICU admission, admission category (medical or surgical), reason for ICU admission (acute exacerbation of COPD, acute respiratory distress syndrome, pneumonia, congestive heart failure, neurologic failure, poisoning, shock [24], cellulitis, or infection), and prior antibiotic use.

During the ICU stay data were collected on tracheostomy, red blood cell transfusion, sedation, stress ulcer prophylaxis, use of neuromuscular-blocking agents, number of days with PaO2 > 120 mmHg, percentage of days with PaO2 > 120 mmHg, occurrence of VAP, duration of mechanical ventilation, and ICU mortality.

### Statistical analysis

SPSS software (SPSS, Chicago, IL, USA) was used for data analysis. Categorical variables were described as frequency (%). The distribution of continuous variables was tested for normality. Normally distributed and skewed continuous variables were described as mean ± standard deviation (SD) or as median and interquartile range (IQR), respectively. All *p* values were two-tailed. Differences were considered significant if *p* values were <0.05.

In order to determine factors associated with VAP, patients with VAP were compared with those without VAP using bivariate and multivariate analyses. The chi-square (χ^2^) test or Fischer’s exact test was used to compare qualitative variables, as appropriate. Student’s *t* test or the Mann-Whitney *U* test was used to compare normally distributed, and skewed continuous variables, respectively. Potential interactions were tested, and the Hosmer-Lemeshow goodness-of-fit was calculated. The odds ratio (OR) and 95 % confidence interval (CI) were calculated for all significant variables in univariate analysis and in multivariate analysis. Exposure to potential risk factors for VAP was taken into account until the occurrence of VAP, or until ICU discharge in patients with and without VAP, respectively.

Because of obvious interaction between hyperoxemia at ICU admission, number of days with hyperoxemia, and percentage of days with hyperoxemia, three different models of multivariate analysis were used. Each of these models included all variables with *p* values <0.1 on univariate analysis, and hyperoxemia at ICU admission, number of days with hyperoxemia, or percentage of days with hyperoxemia. Further, univariable and multivariable Cox proportional hazard models were used to determine risk factors for VAP. All variables with *p* values <0.1 in the univariable model, and hyperoxemia at ICU admission, or percentage of days with hyperoxemia were included in the final multivariable models.

## Results

### Patient characteristics

During the study period 503 patients received mechanical ventilation for >48 h, including 141 patients (28 %) who had at least one episode of VAP. The incidence rate of VAP was 14.7 per 1000 ventilator days. Early-onset and late-onset episodes occurred in 14 (10 %), and 127 (90 %) patients with VAP, respectively. The median (IQR) duration from starting mechanical ventilation to diagnosis of VAP was 14 (8, 23) days.

No significant difference was found in age (median (interquartile range) 60 (49, 73) vs 59 (47, 70), years, *p* = 0.265), SAPS II (48 (35, 61) vs 48 (36, 62), *p* = 0.772), LOD score (5 (2, 7) vs 5 (2, 9), *p* = 0.817), mortality rate (121 out of 289 (41.8 %) vs 82 out of 214 (38.3 %), *p* = 0.265), duration of mechanical ventilation (12 (6, 24) vs 13 (7, 26), *p* = 0.427), and ICU length of stay (15 (8, 29) vs 18 (10, 31), *p* = 0.243), between patients with hyperoxemia and those with no hyperoxemia at ICU admission.

### Risk factors for VAP by univariate analysis

At ICU admission, age, SAPS II, LOD score, and percentage of patients with shock, or with PaO_2_ > 120 mmHg were significantly higher in VAP patients, compared with those with no VAP (Table 1).

**Table 1:** Characteristics of study patients at ICU admission

	VAP		*P*
	Yes	No	
	*n* = 141	*n* = 362	
Age, years	62 (51.5–74)	57 (46–71)	0.004
Male gender	102 (72)	246 (67)	0.339
SAPS II	53 (41–65)	45 (33–59)	<0.001
LOD score	6 (3–9)	5 (2–7)	0.241
McCabe score >2	20 (14)	37 (10)	0.382
Transfer from other wards	96 (68)	216 (60)	0.081
Chronic diseases			
Diabetes	16 (11)	62 (17)	0.108
COPD	41 (29)	98 (27)	0.651
Cardiac failure	34 (24)	67 (18)	0.159
Cirrhosis	4 (3)	14 (4)	0.576
Chronic dialysis	2 (1)	10 (3)	0.375
Immunosuppression	38 (27)	78 (22)	0.196
Admission category			0.499
Medical	91 (65)	245 (67)	
Surgical	48 (34)	114 (31)	
Cause for ICU admission			
Acute exacerbation of COPD	16 (11)	46 (12)	0.677
ARDS	22 (15)	38 (10)	0.113
Pneumonia	40 (28)	91 (25)	0.458
Congestive heart failure	5 (3)	9 (2)	0.516
Neurologic failure	16 (11)	50 (13)	0.462
Poisoning	16 (11)	30 (8)	0.285
Shock	64 (45)	111 (30)	0.002^a^
Cellulitis	10 (7)	48 (13)	0.052
Infection	97 (69)	246 (67)	0.856
Prior antimicrobial treatment	70 (50)	178 (49)	0.924
PaO2 > 120 mmHg	95 (67)	193 (53)	0.004^b^

^a^OR = 1.9, 95 % CI (1.3, 2.8); ^b^OR = 1.8, 95 % CI (1.2, 2.7). The results are expressed in number (%) for categorical variables and in median (IQR) for quantitative variables. *ARDS* acute respiratory distress syndrome, *COPD*, chronic obstructive pulmonary disease, *CI* confidence interval, *ICU* intensive care unit, *LOD* logistic organ dysfunction, *MV* mechanical ventilation, *OR* odds ratio, *PaO*_*2*_ arterial oxygen tension, *SAPS* simplified acute physiology score, *VAP* ventilated-associated pneumonia

During the ICU stay, the percentage of patients with stress ulcer prophylaxis, red blood cell transfusion, or sedation was significantly higher in patients with VAP compared to patients without VAP. Number of days with hyperoxemia was also significantly higher in patients with VAP compared to patients without VAP (Table 2). The percentage of patients with VAP, based on the number of days with hyperoxemia is presented in Fig. 1.

**Table 2:** Patient characteristics during the ICU stay

	VAP		*P*
	Yes	No	
	*n* = 141	*n* = 362	
Stress ulcer prophylaxis			0.002
Proton-pump inhibitor	122 (86)	279 (77)	
Sucralfate	9 (6)	46 (13)	
No	10 (7)	40 (11)	
Tracheostomy	24 (17)	47 (13)	0.243
Red blood cell transfusion	86 (61)	139 (38)	<0.001^a^
Sedation	122 (86)	284 (78)	0.039^b^
Neuromuscular-blocking agent use	10 (7)	20 (6)	0.505
Mean number of ABG per day	3 (1–6)	2 (1–5)	0.261
Number of days with PaO_2_ > 120 mmHg	5 (2–7)	3 (1–5)	<0.001
Percentage of days with PaO_2_ > 120 mmHg	0.33 (0.19–0.58)	0.33 (0.14–0.50)	0.282
Duration of MV prior to VAP, days	14 (8–23)	9 (5–17)	<0.001
Total duration of MV, days	30 (17–43)	9 (5–17)	<0.001
Length of ICU stay, days	34 (19–45.5)	12 (7–21)	<0.001
ICU mortality	73 (52)	130 (35)	0.001^c^

The results are expressed in number (%) for categorical variables and in median (IQR) for quantitative variables. Exposure to potential risk factors for VAP was taken into account until VAP occurrence, or until ICU discharge in patients with and without VAP, respectively. ^a^OR = 2.5 (1.7, 3.7); ^b^OR = 1.8 (1.02, 3.0); ^c^OR = 1.9 (1.3, 2.8)

*VAP* ventilator-associated pneumonia, *CI* confidence interval, *ICU* intensive care unit, *OR* odds ratio, *ABG* arterial blood gas, *PaO*_*2*_ arterial oxygen tension, *MV* mechanical ventilation

### Microbiological results

VAP was polymicrobial in 8 patients (6 %), and related to multidrug resistant (MDR) bacteria in 58 patients (41 %). Gram-negative bacteria represented 78 % of all bacteria, and were identified in 82 % of patients with VAP. *P. aeruginosa* (34 %), *S. aureus* (11 %), and *A. baumannii* (9 %) were the most common bacteria in patients with VAP. MDR bacteria represented 41 % of all bacteria (58 out of 149) (Table 3).

**Table 3:** Microorganisms isolated in patients with ventilator-associated pneumonia

Microorganisms	Number (%)
Gram-negative bacilli	116 (82)
*Pseudomonas aeruginosa*	48 (34)
*Escherichia Coli*	14 (10)
*Acinetobacter baumannii*	13 (9)
Enterobacter sp.	13 (9)
Klebsiella sp.	8 (5)
*Proteus mirabilis*	6 (4)
Serratia sp.	5 (3.5)
*Stenotrophomonas maltophilia*	4 (2.8)
Others	5 (3.5)
Gram-positive cocci	33 (23)
Methicillin-resistant *Staphylococcus aureus*	11 (7.8)
*Staphylococcus epidermidis*	7 (5)
Methicillin-sensitive *Staphylococcus aureus*	5 (3.5)
Enterococcus sp.	5 (3.5)
*Streptococcus pneumoniae*	5 (3.3)

### Risk factors for VAP by multivariate analysis

Because of significant (*p* < 0.05) interactions between hyperoxemia at ICU admission, number of days with hyperoxemia, and percentage of days with hyperoxemia, three different logistic regression models were used with only one of these factors in each model. No other significant (*p* > 0.1) interactions were found between other factors introduced in the multivariable models.

The results of the three logistic regression models are presented in Table 4. Number of days with hyperoxemia, hyperoxemia at ICU admission, and percentage of days with hyperoxemia were independently associated with VAP in models 1, 2, and 3, respectively.

**Table 4:** Factors associated with ventilator-associated pneumonia by multivariate analysis

Variables	*P*	OR (95 % CI)
Model 1		
SAPS II	0.019	1.01 (1.00, 1.02)*
Red blood cell transfusion	0.009	1.75 (1.14, 2.70)
Proton pump inhibitor use	0.040	1.86 (1.03, 3.39)
Number of days with hyperoxemia	0.001	1.10 (1.04, 1.16)**
Model 2		
SAPS II	0.017	1.01 (1.00, 1.02)*
Red blood cell transfusion	0.009	1.79 (1.16, 2.78)
Proton pump inhibitor use	0.031	1.92 (1.06, 3.50)
MV duration prior to VAP	0.012	1.10 (1.01, 1.04)**
Hyperoxemia at ICU admission	0.004	1.89 (1.23, 2.89)
Model 3		
Red blood cell transfusion	0.018	1.71 (1.10, 2.65)
Proton pump inhibitor use	0.017	2.10 (1.14, 3.79)
MV duration prior to VAP	0.005	1.03 (1.01, 1.04)*
Percentage of days with hyperoxemia	0.029	2.20 (1.08, 4.48)**

*SAPS* simplified acute physiology score; *MV* mechanical ventilation, VAP ventilator-associated pneumonia. Model 1: *per point of SAPS II, **per day with hyperoxemia; Hosmer-Lemshow goodness-of-fit, *p* = 0.62. Other non-significant variables included in the model: transfer from other wards, shock, sedation, and duration of MV prior to VAP. Model 2: *per point of SAPS II, **per day of mechanical ventilation; Hosmer-Lemshow goodness-of-fit, *p* = 0.81. Other non-significant variables included in the model: transfer from other wards, shock, and sedation. Model 3: *per day of mechanical ventilation, **per centile of days with hyperoxemia; Hosmer-Lemshow goodness-of-fit, p = 0.78. Other non-significant variables included in the model: SAPS II, transfer from other wards, shock, and sedation

### Risk factors for VAP by univariate and multivariate Cox proportional hazards models

Hyperoxemia at ICU admission and percentage of days with hyperoxemia were independently associated with VAP, using two different Cox proportional hazards models (Table 5).

**Table 5:** Risk factors for ventilator-associated pneumonia by Cox proportional hazards model

Variable	Univariate analysis		Multivariate analysis (model 1)		Multivariate analysis (model 2)	
	HR (95 % CI)	*P*	HR (95 % CI)	*P*	HR (95 % CI)	*P*
At ICU admission						
LOD score	1.04 (1.01, 1.09)	0.044	-	0.922	-	0.138
Transfer from other wards	0.65 (0.45, 0.93)	0.018	-	0.356	-	0.952
Prior antibiotic treatment	0.67 (0.48, 0.94)	0.021	-	0.483	-	0.184
Neurologic failure	1.82 (1.07, 3.09)	0.027	-	0.111	-	0.197
Poisoning	3.24 (1.9, 5.51)	<0.001	2.49 (1.31, 4.72)	0.005	2.16 (1.14, 4.09)	0.018
COPD	0.55 (0.37, 0.89)	0.003	-	0.063	-	0.065
McCabe score >2	0.76 (0.59, 0.99)	0.042	-	0.169		0.726
PaO2 > 120 mmHg	1.58 (1.11, 2.25)	0.011	NA	NA	1.68 (1.16, 2.42)	0.006
During ICU stay						
Percentage of days with PaO2 > 120 mmHg	5.67 (3.15, 10.20)	<0.001	6.23 (3.26, 11.9)	<0.001	NA	NA

*HR* hazard ratio, *LOD* logistic organ dysfunction, *COPD* chronic obstructive pulmonary disease, *NA* not applicable. *P* > 0.1 by univariate analysis for: age, male gender, simplified acute physiology score II, diabetes, cardiac failure, cirrhosis, chronic dialysis, immunosuppression, all causes of ICU admission except neurologic failure, stress ulcer prophylaxis, tracheostomy, red blood cell transfusion, sedation, and neuromuscular-blocking agent use

## Discussion

Our results suggest that hyperoxemia is an independent risk factor for VAP. To our knowledge, our study is the first to evaluate the relationship between hyperoxemia and VAP. As discussed previously, this result could be explained by the occurrence of HALI, atelectasis, and reduced mucocilliary clearance in patients with hyperoxemia [25–28]. Previous studies reported high rates of VAP in patients with acute respiratory distress syndrome [18, 29]. Further, positive expiratory pressure was identified as a preventive measure against VAP [30], by reducing atelectasis in mechanically ventilated patients [31]. Excess oxygen administration could damage tissues through the production of reactive oxygen species (ROS). In excessive concentrations, ROS-mediated stress can lead to cellular necrosis and apoptosis [32]. Therefore, the process of oxidative stress might promote the development of multi-organ failure [33]. In addition, oxidative stress is responsible for direct damage to biological molecules (DNA oxidation, proteins, lipids and carbohydrates) and indirect injury through the release of cytotoxic products and mutagenic effects of lipid oxidation [16].

Recent animal studies support the role of hyperoxemia in the pathogenesis of VAP. Entezari and colleagues demonstrated that prolonged exposure to hyperoxia can compromise the ability of macrophages, an essential part of innate immunity, to phagocytose *P. aeruginosa* [34]. The same group of investigators reported that hyperoxia results in elevated concentrations of high mobility group box-1 (HMGB1), and mortality in mice infected with *P. aeruginosa* [17]. Treatment of these animals with a neutralizing anti-HMGB1 monoclonal antibody allowed a reduction in bacterial counts, injury, and numbers of neutrophils in the lungs, and an increase in leucocyte phagocytic activity compared with control animals. Another recent animal study suggests that hyperoxia increases mortality in mice with *A. baumannii* pneumonia, and that procysteine improves survival by increasing the phagocytic activity of alveolar macrophages [35].

The incidence of multidrug resistant bacteria was relatively high in patients with VAP. This could be explained by the high percentage of patients with late-onset VAP, prior antibiotic treatment, or COPD, and the high severity of illness at ICU admission. All these factors were previously identified as independent risk factors for multidrug resistant bacteria [36–39].

Our study has several limitations. First, it was a retrospective study performed in a single center. Therefore, our results could not be generalized and further prospective multicenter studies are needed to confirm these findings. However, all data, except those related to hyperoxemia, were prospectively collected. Second, the cutoff used for hyperoxemia (>120 mmHg) was selected based on the current literature [12, 40, 41]. For example, normal PaO_2_ is defined by the British Thoracic Society as between 90 and 110 mmHg for patients under 70 years of age and according to the sea level [42]. However, other studies used a different cutoff (i.e., to PaO_2_ ≥ 300 mmHg) to define hyperoxemia [8, 43, 44]. Because a day with at least one PaO_2_ value >120 mmHg was considered as a day with hyperoxemia, our definition of hyperoxemia might have overestimated the time period of hyperoxemia. Continuous control of pulse oximetry could be more appropriate to accurately determine time spent with hyperoxemia. Third, no information was collected on PaO_2_, FiO_2_, or positive end-expiratory (PEEP) values. Some risk factors, such as head-of-bed elevation and under-inflation of tracheal cuff, were not evaluated in this study. Finally, because of the retrospective design, exploratory investigations, such as measurement of ROS or evaluation of immune function could not be performed.

Two recent small studies [2, 5], one before-after and one randomized controlled study, suggested that a conservative oxygenation strategy might be safe in intubated critically ill patients, compared with a liberal strategy. However, none of these studies evaluated the impact of oxygenation strategy on VAP incidence. Further large randomized controlled trials are required to confirm these data, and to determine the impact of oxygenation strategy on VAP incidence.

## Conclusion

Our results suggest a link between hyperoxemia and VAP. However, further large prospective studies are required to confirm these findings and to evaluate the impact of a conservative oxygen strategy vs a conventional strategy, on the incidence of VAP.

## Key messages

- Hyperoxemia is independently associated with ventilator-associated pneumonia
- This association could be explained by the consequences of hyperoxemia, including acute lung injury, atelectasis, and reduced bacterial clearance
- Further large multicenter studies are required to confirm our results

## Abbreviations

ARDS, acute respiratory distress syndrome; cfu, colony-forming units; CI, confidence interval; COPD, chronic obstructive pulmonary disease; FiO_2_, inspired oxygen fraction; HALI, hyperoxic acute lung injury; ICU, intensive care unit; LOD, logistic organ dysfunction; MDR, multidrug resistant; OR, odds ratio; PaO, arterial oxygen tension; ROS, reactive oxygen species; SAPS, simplified acute physiology score; VAP, ventilated-associated pneumonia

## References

[1] Suzuki S, Eastwood GM, Peck L, Glassford NJ, Bellomo R (2013). Current oxygen management in mechanically ventilated patients: a prospective observational cohort study. J Crit Care..

[2] Suzuki S, Eastwood GM, Glassford NJ, Peck L, Young H, Garcia-Alvarez M, Schneider AG, Bellomo R. Conservative oxygen therapy in mechanically ventilated patients: a pilot before-and-after trial. Crit Care Med. 2014;42:1414–22. 10.1097/CCM.0000000000000219 24561566

[3] Rachmale S, Li G, Wilson G, Malinchoc M, Gajic O (2012). Practice of excessive F(IO(2)) and effect on pulmonary outcomes in mechanically ventilated patients with acute lung injury. Respir Care..

[4] Helmerhorst HJF, Schultz MJ, van der Voort PHJ, Bosman RJ, Juffermans NP, de Wilde RBP, van den Akker-van Marle ME, van Bodegom-Vos L, de Vries M, Eslami S, de Keizer NF, Abu-Hanna A, van Westerloo DJ, de Jonge E. Effectiveness and clinical outcomes of a two-step implementation of conservative oxygenation targets in critically ill patients: a before and after trial. Crit Care Med. 2016;44(3):554-63. 10.1097/CCM.0000000000001461 26562347

[5] Panwar R, Hardie M, Bellomo R, Barrot L, Eastwood GM, Young PJ, Capellier G, Harrigan PWJ, Bailey M. Conservative versus liberal oxygenation targets for mechanically ventilated patients, a pilot multicenter randomized controlled trial. Am J Respir Crit Care Med. 2016;193:43–51. 10.1164/rccm.201505-1019OC 26334785

[6] Cornet AD, Kooter AJ, Peters MJL, Smulders YM (2013). The potential harm of oxygen therapy in medical emergencies. Crit Care..

[7] Farquhar H, Weatherall M, Wijesinghe M, Perrin K, Ranchord A, Simmonds M, Beasley R. Systematic review of studies of the effect of hyperoxia on coronary blood flow. Am Heart J. 2009;158:371–7. 10.1016/j.ahj.2009.05.037 19699859

[8] Kilgannon JH, Jones AE, Shapiro NI, Angelos MG, Milcarek B, Hunter K, Parrillo JE, Trzeciak S. Association between arterial hyperoxia following resuscitation from cardiac arrest and in-hospital mortality. JAMA. 2010;303:2165–71. 10.1001/jama.2010.707 20516417

[9] Stub D, Smith K, Bernard S, Nehme Z, Stephenson M, Bray JE, Cameron P, Barger B, Ellims AH, Taylor AJ, Meredith IT, Kaye DM. AVOID Investigators. Air versus oxygen in ST-segment-elevation myocardial infarction. Circulation. 2015;131:2143-50. 10.1161/CIRCULATIONAHA.114.014494 26002889

[10] Helmerhorst HJF, Roos-Blom M-J, van Westerloo DJ, de Jonge E (2015). Association between arterial hyperoxia and outcome in subsets of critical illness: a systematic review, meta-analysis, and meta-regression of cohort studies. Crit Care Med..

[11] Damiani E, Adrario E, Girardis M, Romano R, Pelaia P, Singer M, Donati A. Arterial hyperoxia and mortality in critically ill patients: a systematic review and meta-analysis. Crit Care. 2014;18:711. 10.1186/s13054-014-0711-x PMC4298955 25532567

[12] Eastwood G, Bellomo R, Bailey M, Taori G, Pilcher D, Young P, Beasley R. Arterial oxygen tension and mortality in mechanically ventilated patients. Intensive Care Med. 2012;38:91–8. 10.1007/s00134-011-2419-6 22127482

[13] Sinclair SE, Altemeier WA, Matute-Bello G, Chi EY (2004). Augmented lung injury due to interaction between hyperoxia and mechanical ventilation. Crit Care Med..

[14] Kallet RH, Matthay MA (2013). Hyperoxic acute lung injury. Respir Care..

[15] Dantzker DR, Wagner PD, West JB (1974). Proceedings: Instability of poorly ventilated lung units during oxygen breathing. J Physiol..

[16] Hafner S, Beloncle F, Koch A, Radermacher P, Asfar P (2015). Hyperoxia in intensive care, emergency, and peri-operative medicine: Dr. Jekyll or Mr. Hyde? A 2015 update. Ann Intensive Care.

[17] Patel VS, Sitapara RA, Gore A, Phan B, Sharma L, Sampat V, Li JH, Yang H, Chavan SS, Wang H, Tracey KJ, Mantell LL. High Mobility Group Box-1 mediates hyperoxia-induced impairment of Pseudomonas aeruginosa clearance and inflammatory lung injury in mice. Am J Respir Cell Mol Biol. 2013;48:280–7. 10.1165/rcmb.2012-0279OC PMC3604087 23087050

[18] Forel J-M, Voillet F, Pulina D, Gacouin A, Perrin G, Barrau K, aber S, Arnal J-M, Fathallah M, Auquier P, Roch A, Azoulay E, Papazian L. Ventilator-associated pneumonia and ICU mortality in severe ARDS patients ventilated according to a lung-protective strategy. Crit Care. 2012;16:R65. 10.1186/cc11312 PMC3681394 22524447

[19] van Kaam AH, Lachmann RA, Herting E, De Jaegere A, van Iwaarden F, Noorduyn LA, Kok JH, Haitsma JJ, Lachmann B. Reducing atelectasis attenuates bacterial growth and translocation in experimental pneumonia. Am J Respir Crit Care Med. 2004;169:1046–53. 10.1164/rccm.200312-1779OC 14977624

[20] Rello J, Lisboa T, Koulenti D (2014). Respiratory infections in patients undergoing mechanical ventilation. Lancet Respir Med..

[21] Nair GB, Niederman MS (2014). Ventilator-associated pneumonia: present understanding and ongoing debates. Intensive Care Med..

[22] Nseir S, Martin-Loeches I (2015). In the name of ventilator-associated pneumonia prevention: lung microbiota blown away by colistin!. Eur Respir J..

[23] Guidelines for the management of adults with hospital-acquired, ventilator-associated, and healthcare-associated pneumonia. Am J Respir Crit Care Med. 2005, 171:388–416. 10.1164/rccm.200405-644ST 15699079

[24] Cecconi M, De Backer D, Antonelli M, Beale R, Bakker J, Hofer C, Jaeschke R, Mebazaa A, Pinsky MR, Teboul JL, Vincent JL, Rhodes A. Consensus on circulatory shock and hemodynamic monitoring. Task force of the European Society of Intensive Care Medicine. Intensive Care Med. 2014;40:1795–815. 10.1007/s00134-014-3525-z PMC4239778 25392034

[25] Budinger GRS, Mutlu GM (2013). Balancing the risks and benefits of oxygen therapy in critically ill adults. Chest..

[26] Crapo JD (1986). Morphologic changes in pulmonary oxygen toxicity. Annu Rev Physiol..

[27] Sackner MA, Landa J, Hirsch J, Zapata A (1975). Pulmonary effects of oxygen breathing. A 6-hour study in normal men. Ann Intern Med.

[28] Fox RB, Hoidal JR, Brown DM, Repine JE (1981). Pulmonary inflammation due to oxygen toxicity: involvement of chemotactic factors and polymorphonuclear leukocytes. Am Rev Respir Dis..

[29] Delclaux C, Roupie E, Blot F, Brochard L, Lemaire F, Brun-Buisson C (1997). Lower respiratory tract colonization and infection during severe acute respiratory distress syndrome: incidence and diagnosis. Am J Respir Crit Care Med.

[30] Manzano F, Fernández-Mondéjar E, Colmenero M, Poyatos ME, Rivera R, Machado J, Catalán I, Artigas A. Positive-end expiratory pressure reduces incidence of ventilator-associated pneumonia in nonhypoxemic patients. Crit Care Med. 2008;36:2225–31. 10.1097/CCM.0b013e31817b8a92 18664777

[31] Aboab J, Jonson B, Kouatchet A, Taille S, Niklason L, Brochard L (2006). Effect of inspired oxygen fraction on alveolar derecruitment in acute respiratory distress syndrome. Intensive Care Med..

[32] Martin DS, Grocott MPW (2013). Oxygen therapy in critical illness: precise control of arterial oxygenation and permissive hypoxemia. Crit Care Med..

[33] Motoyama T, Okamoto K, Kukita I, Hamaguchi M, Kinoshita Y, Ogawa H (2003). Possible role of increased oxidant stress in multiple organ failure after systemic inflammatory response syndrome. Crit Care Med..

[34] Entezari M, Weiss DJ, Sitapara R, Whittaker L, Wargo MJ, Li J, Wang H, Yang H, Sharma L, Phan BD, Javdan M, Chavan SS, Miller EJ, Tracey KJ, Mantell LL. Inhibition of high-mobility group box 1 protein (HMGB1) enhances bacterial clearance and protects against Pseudomonas Aeruginosa pneumonia in cystic fibrosis. Mol Med. 2012;18:477–85. 10.2119/molmed.2012.00024 PMC3356431 22314397

[35] Saito K, Kimura S, Saga T, Misonoo Y, Yoshizawa S, Akasaka Y, Ishii T, Kuwano K, Yamaguchi K, Tateda K. Protective effect of procysteine on Acinetobacter pneumonia in hyperoxic conditions. J Antimicrob Chemother. 2013;68:2305–10. 10.1093/jac/dkt192 23681269

[36] Nseir S, Di Pompeo C, Soubrier S, Delour P, Lenci H, Roussel-Delvallez M, Onimus T, Saulnier F, Mathieu D, Durocher A. First-generation fluoroquinolone use and subsequent emergence of multiple drug-resistant bacteria in the intensive care unit. Crit Care Med. 2005;33:283–9. 10.1097/01.ccm.0000152230.53473.a1 15699829

[37] Nseir S, Hoel J, Grailles G, Soury-Lavergne A, Di Pompeo C, Mathieu D, Durocher A. Remifentanil discontinuation and subsequent intensive care unit-acquired infection: a cohort study. Crit Care. 2009;13:R60. 10.1186/cc7788 PMC2689508 19383164

[38] Martin-Loeches I, Deja M, Koulenti D, Dimopoulos G, Marsh B, Torres A, Niederman MS, Rello J. Potentially resistant microorganisms in intubated patients with hospital-acquired pneumonia: The interaction of ecology, shock and risk factors. Intensive Care Med. 2013;39:672–81. 10.1007/s00134-012-2808-5 23358539

[39] Luna CM, Sarquis S, Niederman MS, Sosa FA, Otaola M, Bailleau N, Vay C a, Famiglietti A, Irrazabal C, Capdevila A a. Is a strategy based on routine endotracheal cultures the best way to prescribe antibiotics in ventilator-associated pneumonia? Chest. 2013;144:63–71. 10.1378/chest.12-1477 23348886

[40] Itagaki T, Nakano Y, Okuda N, Izawa M, Onodera M, Imanaka H, Nishimura M. Hyperoxemia in mechanically ventilated, critically ill subjects: incidence and related factors. Respir Care. 2015;60:335–40. 10.4187/respcare.03451 25389354

[41] de Jonge E, Peelen L, Keijzers PJ, Joore H, de Lange D, van der Voort PHJ, Bosman RJ, de Waal RAL, Wesselink R, de Keizer NF. Association between administered oxygen, arterial partial oxygen pressure and mortality in mechanically ventilated intensive care unit patients. Crit Care. 2008;12:R156. 10.1186/cc7150 PMC2646321 19077208

[42] O’Driscoll BR, Howard LS, Bucknall C, Welham SA, Davison AG (2011). British Thoracic Society emergency oxygen audits. Thorax..

[43] Bellomo R, Bailey M, Eastwood GM, Nichol A, Pilcher D, Hart GK, Reade MC, Egi M, Cooper DJ. Arterial hyperoxia and in-hospital mortality after resuscitation from cardiac arrest. Crit Care. 2011;15:R90. 10.1186/cc10090 PMC3219350 21385416

[44] Rincon F, Kang J, Maltenfort M, Vibbert M, Urtecho J, Athar MK, Jallo J, Pineda CC, Tzeng D, McBride W, Bell R. Association between hyperoxia and mortality after stroke: a multicenter cohort study. Crit Care Med. 2014;42:387–96. 10.1097/CCM.0b013e3182a27732 24164953

